# Crashes involving motorised rickshaws in urban India: Characteristics and injury patterns

**DOI:** 10.1016/j.injury.2009.10.049

**Published:** 2011-01

**Authors:** Uli Schmucker, Rakhi Dandona, G. Anil Kumar, Lalit Dandona

**Affiliations:** a George Institute for International Health - India, Hyderabad, India; b George Institute for International Health and School of Public Health, University of Sydney, Sydney, Australia; c Department of Trauma and Orthopaedic Surgery/Traffic Crash Research Unit, Ernst-Moritz-Arndt-University of Greifswald, Sauerbruchstrasse, Germany; d Public Health Foundation of India, New Delhi, India; e Administrative Staff College of India, Hyderabad, India; f Institute for Health Metrics and Evaluation, University of Washington, Seattle, USA

**Keywords:** Auto, Rickshaw, India, Injuries, Road traffic injuries, Three-wheeled vehicle, Vulnerable road user

## Abstract

**Introduction:**

Motorised three-wheeled vehicles (motorised rickshaw) are popular in Asian countries including India. This study aims to describe the crash characteristics and injury patterns for motorised rickshaw occupants and the road users hit-by-motorised rickshaw in urban India.

**Methods:**

Consecutive cases of road traffic crashes involving motorised rickshaw, irrespective of injury severity, whether alive or dead, presenting to the emergency departments of two large government hospitals and three branches of a private hospital in Hyderabad city were recruited. Crash characteristics, details of injuries, injury severity parameters and outcome were documented in detailed interviews.

**Results:**

A total of 139 (18%) of the 781 participants recruited were injured as a motorised rickshaw occupant (11%) or were hit by a motorised rickshaw (7%) in 114 crashes involving motorised rickshaw. Amongst motorised rickshaw occupants, single-vehicle collisions (54%) were more frequent than multi-vehicle collisions (46%), with overturning of motorised rickshaw in 73% of the single-vehicle collisions. Mortality (12%), the mean Injury Severity Score (5.8) and rate of multiple injured (60%) indicated a substantial trauma load. No significant differences in injury pattern were found between motorised rickshaw occupants and hit-by-motorised rickshaw subjects, with the pattern being similar to that of the pedestrians and two-wheeled vehicle users. With bivariate analysis for motorised rickshaw occupants, the risk of fatal outcome (odds ratio (OR) 2.60, 95% confidence interval (CI): 0.64–10.54), upper limb injury (OR 2.25, 95% CI: 0.94–5.37) and multiple injuries (OR 2.03, 95% CI 0.85–4.83) was high, although not statistically significant in multi-motorised-vehicle collisions as compared with the single-vehicle collisions or overturning. The risk of having multiple injuries (OR 4.55, 95% CI: 1.15–17.95) was significantly higher in motorised rickshaw occupants involved in front collisions. Being a front-seat motorised rickshaw passenger in a vehicle collision increased the risk of having a fatal outcome (OR 7.37, 95% CI: 0.83–65.66) and a Glasgow coma score ≤ 12 (OR 2.21, 95% CI: 0.49–9.89), although not significantly when compared to the back-seat passengers.

**Conclusion:**

These findings can assist with planning to deal with the consequences and prevention of road traffic injuries due to crashes involving motorised rickshaw, given the high use of these and substantial morbidity of related injuries in India. The need for improved understanding of the risk characteristics of motorised rickshaw is highlighted.

## Introduction

With approximately 105 000 people killed and 453 000 injured in road traffic crashes (RTCs) in India in 2006,^25^ RTCs pose a major burden to the health system, crash victims and their families. Though India accounts for the majority of injuries and fatalities due to RTCs in the Southeast Asian region,^26^ only 0.1% of health research published from India in 2002 had addressed RTCs.^6^ Previous studies from India have reported on vulnerable road users who are at a higher risk for crash involvement, injury and death.^10–12,16,17,22,25,26,33^

**Fig. 1:**
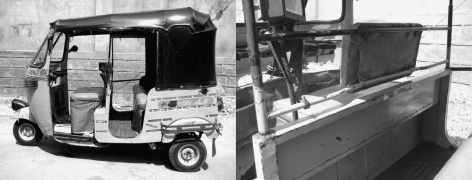
Motorised rickshaw: side view (left) and view from the passenger entrance (right). Note the metal cabin wall and rigid crossbars which can result in an injury in a road traffic crash.

Motorised three-wheeled vehicles (motorised rickshaws also popularly known as autos, Fig. 1) have a capacity to seat three adults or six children in addition to the driver. These are an important means of public transport and its variations are quite popular across the south and east Asia. Being lightweight, flexible vehicles and available in hybrid and gas-powered versions, motorised rickshaws are a potential vehicle of the future, especially in congested traffic of mega cities. However, biomechanical studies have revealed the limited crashworthiness and serious injury risk even at a low crash speed for motorised rickshaw occupants and pedestrians hit-by-motorised rickshaws.^5,23^ In addition, motorised rickshaw drivers are known for hazardous driving practices and are documented to be amongst the major violators of traffic laws.^9^ This likely implies a substantial injury risk for those exposed to motorised rickshaws, including the other road users. We have recently reported the annual incidence rate for non-fatal road traffic injuries (RTIs) for the users of motorised rickshaws aged 5–49 years at 0.23% from the Indian city of Hyderabad.^10^ The National Crime Records Bureau of India reported that motorised rickshaws comprised 5.6% of all RTC deaths in India, hence approximating 5900 deaths in 2006.^25^ One-third of these deaths were reported from the Indian state of Andhra Pradesh.^25^

Crashes involving motorised rickshaws and the resulting injury patterns have not been studied yet in India. The objective of this study was to analyse crash patterns involving motorised rickshaws and the resulting injuries in Hyderabad city in India. This analysis could assist in understanding the causation of crash and trauma, which will have important implications for the identification of targeted prevention measures.

## Materials and methods

The setting for the study was Hyderabad city, capital of the Andhra Pradesh state in India, with an estimated population of 3.8 million in 2001.^27^ Hyderabad had 63 746 motorised rickshaws on road, accounting for 56% of all contract carriages registered in 2001–2002.^20^ Amongst all the registered vehicles, motorised rickshaws were third only to motorised two-wheeled vehicles and cars.^20^ This study was approved by the Ethics Committee of the Administrative Staff College of India, Hyderabad, India.

The methods for this study have been described previously.^16^ Between November 2005 and June 2006, consecutive subjects reporting to two large public hospitals and three branches of a large private hospital in Hyderabad due to RTCs were recruited for this study. Irrespective of age, injury severity or outcome, all RTC subjects who reported to the emergency department alive or brought dead were included. RTI was defined as any injury resulting from RTCs. Trained field staff was posted around-the-clock in the emergency departments and respective mortuaries to document all consecutive RTI cases. A questionnaire was used by trained staff for detailed interview after obtaining written informed consent from the injured person or the caretaker, or a responsible adult family member in fatal cases. Data were collected from the injured person where possible, or the caretaker or adult family member where this was not possible. Detailed data on the demographics of those injured, characteristics of the crash, Glasgow coma score (GCS) on arrival at hospital, details of injuries sustained and final disposition were documented.

Details of injuries were completed by the physician in the emergency department who attended to the particular RTC case. For those who died at the scene or *en route* to the hospital, the physician attached to the hospital mortuary completed the injury documentation. The injuries were noted in detail and were later classified according to broad categories as per the International Statistical Classification of Diseases and Related Health Problems Version 10 (ICD-10),^34^ (Maximum) Abbreviated Injury Scale ((M)AIS)^2^ and Injury Severity Score (ISS)^3^ by US.

Data were entered into an MS Access database. Analyses were conducted using the SPSS software package, version 16 (SPSS Inc., Chicago, IL, USA), and statistical significance was set at *p* ≤ 0.05. The main outcomes reported are the crash characteristics, injury patterns and outcomes amongst the drivers and passengers of motorised rickshaw (henceforth referred to as motorised rickshaw occupants) and the other type of road users injured due to a crash involving a motorised rickshaws (henceforth referred to as hit-by-motorised rickshaw subjects). Mean value, standard deviation (SD) and range are presented where appropriate. Principal comparisons of resulting injury pattern and severity parameters between motorised rickshaw occupants and hit-by-motorised rickshaw subjects were made using the Chi-square test. Bivariate analysis was performed to assess the risk of certain injury parameters in the two groups combined. Mean values of numbers of occupants and numbers of injured were compared by independent sample *t*-test. Direction of the collision impact is reported for the motorised rickshaw occupant cases. Further bivariate analysis was performed to assess the risk of certain injury parameters amongst motorised rickshaw occupants for a multi-motorised vehicle crash (those involving at least another motorised vehicle in addition to the motorised rickshaw), a crash with vehicle-front impact and for a front-seat passenger in a motorised rickshaw during vehicle collision.

## Results

**Fig. 2:**
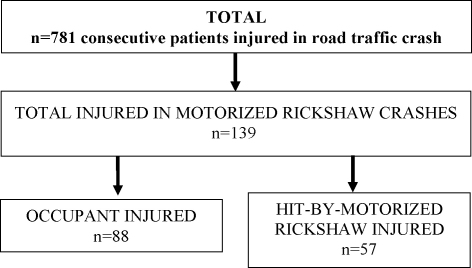
Selection of cases involving motorised rickshaws and study subjects injured in motorised rickshaw crashes. Occupant includes motorised rickshaw driver and passenger.

A total of 781 consecutive RTI cases were recruited in the study; of these, 139 subjects (18%) were injured in 114 crashes involving motorised rickshaws (Fig. 2). Amongst the 114 crashes involving motorised rickshaws, in 68 (60%) cases a motorised rickshaw occupant was injured and 52 (46%) cases were hit-by-motorised rickshaw subjects, with an overlap of six subjects who were hit by a motorised rickshaw while being a passenger in another motorised rickshaw. Amongst these 139 injured subjects, 88 (66%) were motorised rickshaw occupants and 57 (41%) were hit-by-motorised rickshaw subjects (Table 1).

### Crash characteristics

Table 1 presents the crash characteristics. The 114 motorised rickshaw crashes comprised 52 (46%) multi-motorised vehicle crashes with 71 injured (52%), and 62 (54%) single-motorised vehicle crashes with 68 injured (48%, crashes in which the motorised rickshaw was the only motorised vehicle involved). Motorised two-wheeled vehicles were the most frequently involved other vehicles in multi-motorised vehicle crashes (24 cases, 46%) while overturning was the most frequent crash mechanism in single-motorised vehicle crashes (27 cases, 44%).

At the time of the crash, passengers were being carried in 50 motorised rickshaw occupant cases (74%) with the mean number of passengers being 4.1 (SD 2.7, range 1–15 passengers). Overloading with more than three passengers was found in 25 cases (50% of cases in which passengers were carried). Four to six passengers were on board in 18 (36%) cases and 7–15 passengers in 7 (14%) cases. The number of injured or dead passengers (irrespective of inclusion in this study, because not all the injured/dead in each crash were brought to the hospitals participating in the study) was found to be significantly higher in motorised rickshaws with more than three passengers (mean 4.5 injured or dead per case, standard deviation (SD) 3.5) than in those with three or less passengers (mean 1.8, SD 1.0; *p* < 0.001). Motorised rickshaws that overturned without a primary collision were loaded with a mean of 3.6 passengers (SD 2.9, range 1–12), which was not significantly different from the other motorised rickshaw occupant cases (*p* > 0.05).

Distribution of time of crash for motorised rickshaw crashes involving collision with pedestrians/cyclists/motorised two-wheeled vehicles showed a significant peak in the evening and early night hours (16:30–22:30 h, 49%). The same peak also applied for multi-motorised vehicle crashes (47%). In single-motorised vehicle crashes, the crashes due to driving into a ditch/hole occurred in darkness but no determined peak time was seen for either overturned motorised rickshaw or those which hit an object, or the case involving falling off a motorised rickshaw. The motorised rickshaw-to-pedestrian crashes (*n* = 21, 18%) showed an evening peak (18:01–00:00 h, 43%) with no crashes between midnight and 06:00 h. The collisions occurred with pedestrians while they were walking on the road (*n* = 8), crossing away from a pedestrian crossing (*n* = 6) or just standing on the road (*n* = 4). One case each comprised ‘working on the road’ and ‘stopping a motorised rickshaw for hire’ at the time of crash.

### Injured subjects’ characteristics

Of the 32 motorised rickshaw drivers injured as motorised rickshaw occupants, all were males with a mean age of 28.6 years (SD 8.5, range 14–45 years) with two drivers being under the age of 16 years and 50% aged between 20 and 29 years. Eight drivers (25%) had no valid driving licence for a motorised rickshaw and eight (25%) had self-reported to be under the influence of alcohol at the time of crash. No samples to quantify the blood alcohol concentration were obtained in the emergency departments.

Amongst 56 motorised rickshaw passengers, 36 (64%) were males (Chi-square (*χ*^2^) = 4.6, *p* = 0.033) and the mean age was 31.8 years (SD 15.7, range 1–70 years). Half of the passengers were aged 20–39 years. Ten (18%) passengers reported sitting on the left or right side of the driver's seat, 13 (23%) were seated on the left side on the passenger seat (open side to enter into the motorised rickshaw), eight (14%) in mid-position and eight (14%) on the right side (closed side with a horizontal rod) (schematic diagram of motorised rickshaw shown in Fig. 2). Five (9%) passengers were using the student bench, which is a board of approximately 15 cm width fixed to the back of the driver's cabin separator (to increase seating capacity for children). However, only one of these five occupants was a child.

Amongst 57 hit-by-motorised rickshaw subjects, 46 (81%) were males (*χ*^2^ = 21.5, *p* < 0.001) and the mean age was 34.8 years (SD 16.7, range: 5–80 years) with a similar age distribution as the motorised rickshaw passengers. Most subjects were involved as non-motorised road users (27 subjects, 47%; of which 24 were pedestrians, 89%) or motorised two-wheeled vehicle users (23 subjects, 40%) followed by motorised rickshaw occupants (six subjects, 11%) and a car occupant (one subject, 2%).

### Injury pattern and severity

Injury severity parameters are presented in Table 2. Eleven (13%) of the 88 injured motorised rickshaw occupants and seven (12%) of 57 hit-by-motorised rickshaw subjects died. Four (5%) of the injured motorised rickshaw occupants died on the spot and none in the other group. There were no significant differences between the two groups for the variables presented (*χ*^2^-test, *p* > 0.05) including the overall fatality–survival outcome. The mean ISS (ISS 5.8, SD 10.9) of motorised rickshaw occupants (ISS 6.2, SD 11.2) and hit-by-motorised rickshaw subjects (ISS 5.3, SD 10.0) indicates a significant potential of injury in motorised rickshaw crashes (Table 3). No significant differences between the groups were found with regard to ISS (*p* > 0.05) or MAIS of particular body regions (*χ*^2^-tests, all *p* > 0.05).

Table 4 presents an overview of the injury pattern. Amongst the motorised rickshaw occupants, minor injuries (e.g., bone contusions and superficial wounds) and injuries to the head and neck were more frequent than in any other body region (*χ*^2^-tests, all *p* ≤ 0.006). In hit-by-motorised rickshaw subjects, there was no significant difference between frequency of minor head, neck and limb injuries; however, these were more frequent than other injuries (*χ*^2^-tests, all *p* ≤ 0.024). In both groups, fractures, crush, intracranial or organ injuries were recorded more frequently in the lower limb region (*χ*^2^-tests, all *p* ≤ 0.020) than in the head, neck or body stem. No significant difference was found between injuries to the lower and upper limbs. The proportions of injuries other than minor injuries amongst all injuries per body region were significantly higher in limb trauma (those being not life-threatening) than in trauma of the head, neck or body stem (those being a potential threat to life) (*χ*^2^ = 10.7, *p* = 0.001). No statistically significant differences were found in the injury patterns between the two groups (*χ*^2^-tests, all *p* > 0.05).

Lower limb fractures were the most frequent specific injuries in both groups with mostly femoral fractures in motorised rickshaw occupants (nine femoral, seven tibial/fibular and four foot) and tibial/fibular fractures in hit-by-motorised rickshaw subjects (eight tibial and one femoral). Multiple injuries were documented in 53 (63%) motorised rickshaw occupants and 32 (59%) hit-by-motorised rickshaw subjects. In subjects with multiple injuries in both groups, body stem was significantly less affected than limbs, head and neck (*χ*^2^-tests, all *p* ≤ 0.012) (Table 2). With bivariate analysis, the risk of having a fatal outcome (odds ratio (OR) 0.35, 95% confidence interval (CI): 0.07–1.77), multiple injuries (OR 0.41, 95% CI: 0.16–1.05), upper limb injury (OR 0.41, 95% CI: 0.16–1.08) and GCS ≤ 12 (OR 0.41, 95% CI: 0.10–1.64) was lower in overturning of vehicles than in multi-motorised vehicle collisions though not significant. No significant association was found for lower limb (OR 0.68, 95% CI: 0.27–1.71), head (OR 0.77, 95% CI: 0.30–2.00) or body stem injuries (OR 1.22, 95% CI: 0.38–3.94).

**Fig. 3:**
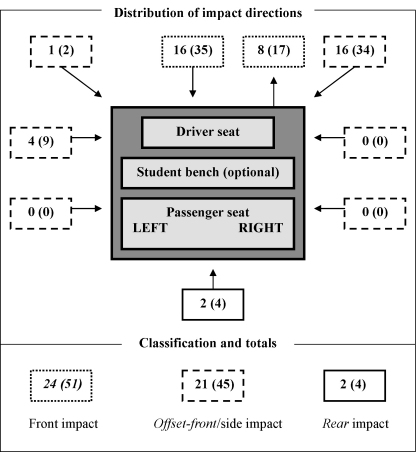
Distribution and classification of vehicle impact direction for motorised rickshaw occupants who were injured in collision with a motorised vehicle (*n* = 47) [number (%)]. The grey area is schematic representation of a motorised rickshaw.

On considering the 88 motorised rickshaw occupant cases using bivariate analysis (Table 5), an increased risk, although not statistically significant of having a fatal outcome (OR 2.60, 95% CI: 0.64–10.54) or upper limb injury (OR 2.25, 95% CI: 0.94–5.37), was found for multi-motorised vehicle collisions when compared with single-vehicle collisions and overturning of vehicles. The impact zone (where the collision occurred) on the motorised rickshaw was recorded for all crashes involving collision with a motorised vehicle. Fig. 3 illustrates the distribution and classification of impact zones for those 47 motorised rickshaw occupants. In general, front and offset-front/side impacts were the leading impact zones (*χ*^2^ = 27.0, *p* < 0.001) with significantly more impacts recorded as an offset-front or side impact (*χ*^2^ = 5.8, *p* = 0.016). Impacts to the front, offset-front and side of the driver seat were more frequent than the rear impacts or side impacts to the passenger seat (*χ*^2^ = 40.3, *p* < 0.001). A significantly higher risk for sustaining multiple injuries (OR 4.55, 95% CI: 1.15–17.95) was found in front impacts of a motorised rickshaw to a vehicle than in the offset-front and side impacts (Table 5). An increased risk of having a fatal outcome or sustaining lower limb injuries, although not statistically significant, was seen in front-seat motorised rickshaw occupants in vehicle collisions as compared with back-seat and student-bench occupants (Table 5).

## Discussion

This study presents real crash and injury data amongst a cohort of motorised rickshaw occupants and road users hit-by-motorised rickshaws. Principal results include the significant injury severity, and importantly, the injury pattern of motorised rickshaw occupants being comparable with that of the pedestrians and motorised two-wheeled vehicle users. Collision with another vehicle was the dominating crash mechanism and overloading of motorised rickshaws and overturning were also frequent.

Motorised rickshaws are amongst the most frequent contract carriages on road in India, including Andhra Pradesh.^20,25^ However, there is little research output related to RTIs and motorised rickshaws.^18,21,26^ This is in contrast to the otherwise relatively strong research focus on vulnerable road users such as pedestrians and motorised two-wheeled vehicle users in Asia.^8,11,16,18,24,26^ The prospective data collection of consecutive crashes irrespective of the severity and outcome from public and private hospitals is the strength of this study. This allows for a broad overview on the key aspects of crashes involving a specific vehicle. However, these data are limited to those seeking care in the study hospitals and the analysis is limited due to small sub-samples with these crashes not being representative of all the crashes involving motorised rickshaws in Hyderabad. It is possible that some injury diagnoses may have been missed since we relied on the hospital physicians for the completion of injury patterns in the questionnaire. Substantial rate of missed diagnoses has been reported previously in an Indian cohort of trauma deaths.^32^

Amongst the motorised rickshaw occupant cases, collision partners in multi-vehicle collisions tended to be light motor vehicles, trucks or buses. This pattern is well known for vulnerable road users.^1,11,18^ Single-vehicle crashes were dominated by overturning of motorised rickshaw, and this has not been described previously. Although the causation of overturning was not examined in this study, driving speed, road-related factors (e.g., road bumps and holes) and vehicle-related factors could be the possible contributors. A strong influence of vehicle design on the stability of motorised rickshaw, for example, when driving over bumps or similar road irregularities, has been documented, including wheel lift-offs when driving a motorised rickshaw straight over road bumps in an experimental setting.^19^ With one-third of all injuries amongst the motorised rickshaw occupants resulting from overturning of the motorised rickshaw, it is imperative that the stability of motorised rickshaw is looked into further detail to prevent these injuries.

Risky driving behaviour and low compliance with traffic regulations have been reported from India and other Asian countries.^1,4,8,14,16,18,26^ This study identified self-reported alcohol consumption in 25% of motorised rickshaw drivers, and 25% unlicensed motorised rickshaw drivers amongst all the motorised rickshaw cases, which is comparable to that reported earlier for other road users.^8,18,26^ A study from Sri Lanka has also reported substantial proportions of motorised rickshaw drivers without valid driver's license.^14^ Interestingly, we found frequent non-compliance of regulations by the motorised rickshaw passengers as well, including misuse of the student bench by adults, passengers not sitting on the designated passenger seat and overloading of passengers. Our findings support the need for stricter enforcement of traffic laws amongst motorised rickshaw drivers, especially because they have been documented to be amongst the main traffic violators in Hyderabad.^9^ As an individual motorised rickshaw driver may be limited in his scope for action, appropriate involvement of relevant stakeholders including the motorised rickshaw driver unions and motorised rickshaw fleet owners would be necessary to improve road safety amongst these drivers. In addition, these data suggest the need to explore means to improve compliance for road safety amongst the motorised rickshaw passengers to reduce their risk of RTIs.

A trend towards an increased risk for injury to most body regions and fatal outcome was found to be associated with multi-motorised vehicle collisions than with single-motorised vehicle collisions or overturning of a motorised rickshaw. Mortality rates were comparable to those reported for vulnerable road users.^16,18,26^ The distribution of injuries with a high proportion of fractures and crush injuries of the limbs in hit-by-motorised rickshaw subjects, who included pedestrians, cyclists and motorised two-wheeled vehicle users was typical of that for vulnerable road users.^1,11,13,15,17,31^ Most of the limb injuries were located in the lower thigh region, which may have resulted from initial impact of the motorised rickshaw front.^5^ Interestingly, the injury pattern of motorised rickshaw occupants was also rather typical of that for pedestrians and motorised two-wheeled vehicle users than for occupants of other types of vehicles.^1,13,15,16,26,31^ The high proportion of lower limb fractures and crush injuries is noticeable, which reflects the high lower limb impact severity that has been previously documented in biomechanical impact simulations.^5,23^ Lower limb injuries to car occupants have also been explained by an intrusion mechanism.^30^ In biomechanical models of frontal bus-to-motorised rickshaw impact at 30 km h^−1^, deformation of more than 30 cm was found at the motorised rickshaw front.^23^

It is noteworthy that no significant difference of injury risk from vehicle collision was found between front-seat and back-seat/student-bench motorised rickshaw occupants, although front and offset-front impact clearly dominated. Real-world injury data from this study support the results of previous impact simulations,^5,23^ which found higher impact velocities, accelerations and peak impact forces of a backseat passenger than those of a driver even in low-speed front impacts. Maximum forces were measured for a passenger's knee-to-cabin separator contact in motorised rickshaw crashes, which is different from advanced vehicles with designated safety devices, where increased injury risk is shown close to the impactor or deformation zone.^28–30^ Although this study was not designed to capture data on the deformation of the motorised rickshaw or injury causation, these data indicate that the crashworthiness and safety standards of motorised rickshaw are questionable. In this context it has been suggested previously that major modifications (e.g., changing the seat orientation of motorised rickshaw passenger from forward to backward and use of motorised rickshaw seat belts) as well as minor changes (e.g., padding of stiff surfaces) have the potential to reduce impact forces of an occupant's body against vehicle parts.^5,23^ Future research must include systematic vehicle-based investigations alongside in-depth analyses at the crash scene to determine the potential to reduce crash and injury risk by appropriate measures.

Road safety in India is predominately based upon police documentation systems, although these have substantial limitations.^7^ This study can serve as a reference for further research, to help identify research priorities, and to assist in traffic planning and vehicle safety legislations for motorised rickshaw as such data for India are not readily available.

## Conclusion

These data on crashes and injuries sustained in crashes involving motorised rickshaws can assist with planning to deal with the consequences as well as prevention of RTIs given the high use of motorised rickshaws and substantial morbidity of related injuries in India. Improved understanding of the risk characteristics of motorised rickshaws is needed to develop safer motorised rickshaws for mega cities.

## Conflict of interest

None.

## Funding

This study was supported by the Wellcome Trust, UK (077002/Z/05/Z). The Wellcome Trust had no involvement in the study design; collection, analysis and interpretation of data; the writing of the article; the decision to submit the article for publication. R. Dandona is supported in part by the National Health and Medical Research Council Capacity Building Grant in Injury Prevention and Trauma Care, Australia. U. Schmucker is supported by a scholarship of the Alfried Krupp von Bohlen und Halbach Foundation, Germany.

## Authors’ contributions

US planned and led the drafting of the article in conjunction with RD, coded the injuries assisted by GAK and analysed data. RD conceptualised and designed the study, GAK managed the data and LD contributed to the design of the study. Interpretation of findings, drafting of the article and final review were performed jointly by the authors.

## Figures and Tables

**Table 1:** Injury severity by crash mechanism for motorised rickshaw occupants and hit-by-motorised rickshaw subjects.

Motorised rickshaw occupant	Number of crashes	Subjects fatally injured	Subjects with multiple injuries	All injured subjects
	*n* = 68, *n* (%)	*n* = 11, *n* (%)	*n* = 53, *n* (%)	*n* = 88, *n* (%)
*Cause of crash*				
Collision with another vehicle/object	37 (54)	9 (82)	37 (70)	54 (61)
Truck/lorry/bus^a^	11 (16)	4 (36)	20 (38)	25 (28)
Motorised two-wheeled vehicle^a^	3 (4)	0	2 (4)	3 (3)
Pedestrian/bicycle^b^	1 (2)	0	0	1 (1)
Car/jeep/van/motorised rickshaw^a^	16 (24)	4 (36)	10 (19)	19 (22)
Object^b^	6 (9)	1 (9)	5 (9)	6 (7)
Other than collision	31 (46)	2 (18)	16 (30)	34 (39)
Overturn^b^	27 (40)	2 (18)	14 (26)	29 (33)
Fell off the motorised rickshaw^b^	2 (3)	0	1 (2)	2 (2)
Ditch/hole^b^	2 (3)	0	1 (2)	3 (3)

Hit-by-motorised rickshaw	Number of crashes	Subjects fatally injured	Subjects with multiple injuries	All injured subjects
	*n* = 52, *n* (%)	*n* = 7, *n* (%)	*n* = 32, *n* (%)	*n* = 57, *n* (%)
*Type of road user*				
Pedestrian/cyclist	24 (46)	3 (43)	14 (44)	27 (47)
Motorised two-wheeled vehicle	21 (40)	3 (43)	15 (47)	23 (40)
Motorised rickshaw/car	7 (14)	1 (14)	3 (9)	7 (12)

a Multi-motorised vehicle crash.

b Single motorised vehicle crash.

**Table 2:** Injury severity and outcome parameters in motorised rickshaw occupants (*n* = 82) and hit-by-motorised rickshaw subjects (*n* = 51).

	Motorised rickshaw occupants, *n* = 82 (%)^a^	Hit-by-motorised rickshaw subjects, *n* = 51 (%)^a^
Mortality		
Died on the spot	4 (5)	0
Died en route	1 (1)	1 (2.0)
Died in hospital	5 (6)	5 (10)

Patient post-crash status		
Conscious	63 (72)	37 (65)
Unconscious but alive	19 (23)	18 (35)
Died on the spot	4 (5)	0

Whole body Maximum Abbreviated Injury Scale^2^		
MAIS 1	36 (44)	29 (57)
MAIS 2–3	43 (52)	21 (41)
MAIS 4–5	1 (1)	0
MAIS 6	2 (2)	1 (2)

Multiple injuries		
Yes, including head injury	41 (50)	19 (37)
Yes, including upper limb injury	30 (37)	21 (41)
Yes, including chest abdomen, pelvis injury	15 (18)	5 (10)
Yes, including lower limb injury	31 (38)	20 (39)
No multiple injuries	31 (38)	21 (41)

Glasgow coma score on arrival at hospital		
13–15	67 (82)	41 (80)
9–12	4 (5)	4 (8)
3–8	6 (7)	5 (10)
Dead on arrival	5 (6)	1 (2)

a After exclusion of 6 subjects who were in motorised rickshaw when hit by another motorised rickshaw.

**Table 3:** Injury pattern of motorised rickshaw occupants (*n* = 82) and hit-by-motorised rickshaw subjects (*n* = 51) by Maximum Abbreviated Injury Scale and body region^2^.

Maximum Abbreviated Injury Scale region	MAIS 0–1, *n* (%)	MAIS 2–3, *n* (%)	MAIS 4–5, *n* (%)	MAIS 6, *n* (%)	MAIS 9, *n* (%)
Motorised rickshaw occupants, *n* = 82^a^					
Head, neck	74 (90)	4 (5)	0	2 (2)	2 (2)
Face	78 (95)	4 (5)	0	0	0
Chest	81 (99)	1 (1)	0	0	0
Abdominal, pelvic contents	80 (98)	2 (2)	0	0	0
Extremities, pelvic girdle	47 (57)	34 (41)	1 (1)	0	0

Hit-by-motorised rickshaw subjects, *n* = 51^a^					
Head, neck	49 (96)	0	0	1 (2)	1 (2)
Face	48 (94)	2 (4)	0	0	1 (2)
Chest	50 (98)	0	0	0	1 (2)
Abdominal, pelvic contents	51 (100)	0	0	0	0
Extremities, pelvic girdle	32 (63)	19 (37)	0	0	0

a After exclusion of 6 subjects who were in motorised rickshaw when hit by another motorised rickshaw.

**Table 4:** Injury pattern of motorised rickshaw occupants (*n* = 82) and hit-by-motorised rickshaw subjects (*n* = 51) by International Statistical Classification of Diseases and related health problems Version 10.

ICD-10 body region^a^	Motorised rickshaw occupants, *n* = 82, *n* (%)	Hit-by-motorised rickshaw subjects, *n* = 51 *n* (%)
Head and neck		
Superficial, open wound	43 (52)	23 (45)
Fracture	4 (5)	2 (4)
Crush, intracranial injury	4 (5)	1 (2)
Other, unspecified	4 (5)	1 (2)

Thorax, abdomen, pelvis		
Superficial, open wound	12 (15)	7 (14)
Fracture	4 (5)	0
Organ injury	2 (2)	0
Other, unspecified	0	2 (4)

Upper extremity		
Superficial, open wound, sprain, contusion	23 (28)	18 (35)
Fracture	12 (15)	4 (8)
Crush injury	2 (2)	2 (4)

Lower extremity		
Superficial, open wound, sprain, contusion	18 (22)	13 (25)
Fracture	20 (24)	9 (18)
Crush injury	6 (7)	3 (6)
Other, unspecified	1 (1)	2 (4)

a ICD-10: International Statistical Classification of Diseases and related health problems Version 10.^34^

**Table 5:** Predictors for the risk of select injury variables by bivariate analysis for motorised rickshaw occupants in a multi-motorised vehicle collision, a crash with vehicle-front impact in a multi-motorised vehicle collision, and as a front-seat passenger in a motorised rickshaw in a multi-motorised vehicle collision.

Motorised rickshaw occupants		Number	Multi-motorised vehicle collision, *n* (% of number)	Odds ratio, referent: single-vehicle collision/overturning (95% CI^a^)
*Region of injury of 88* motorised rickshaw *occupants in multi-motorised or single-vehicle collision*				
Head/neck	Yes	53	31 (58)	1.67 (0.71–3.96)
	No	35	16 (46)	

Upper limb	Yes	37	24 (65)	2.25 (0.94–5.37)
	No	51	23 (45)	

Body stem	Yes	17	8 (47)	0.73 (0.25–2.11)
	No	71	39 (55)	

Lower limb	Yes	47	28 (60)	1.71 (0.73–3.98)
	No	41	19 (46)	

Severity parameters				
Multiple injuries	Yes	53	32 (60)	2.03 (0.85–4.83)
	No	35	15 (43)	

Glasgow coma score ≤12	Yes	16	10 (63)	1.58 (0.52–4.80)
	No	72	37 (49)	

Fatal outcome	Yes	11	8 (73)	2.60 (0.64–10.54)
	No	77	39 (51)	

Motorised rickshaw occupants		Number	Front impact *n* (% of number)	Odds ratio, referent: *offset-front*/side impact (95% CI^a^)
*Region of injury in 45 subjects involved in front, offset-front or side impacts*				
Head/neck	Yes	30	18 (58)	2.25 (0.64–7.97)
	No	15	6 (40)	

Upper limb	Yes	22	12 (55)	1.10 (0.34–3.55)
	No	23	12 (52)	

Body stem	Yes	8	5 (63)	1.58 (0.33–7.59)
	No	37	19 (51)	

Lower limb	Yes	27	17 (63)	2.67 (0.78–9.12)
	No	18	7 (39)	

Severity parameters				
Multiple injuries	Yes	31	20 (65)	4.55 (1.15–17.95)
	No	14	4 (29)	

Glasgow coma score ≤12	Yes	10	6 (60)	1.42 (0.34–5.91)
	No	35	18 (51)	

Fatal outcome	Yes	8	5 (63)	1.58 (0.33–7.59)
	No	37	19 (51)	

Motorised rickshaw occupants		Number	Front seat, *n* (% of number)	Odds ratio, referent: back seat (95% CI^a^)
*Region of injury in 47 front or back-seat subjects in multi-motorised vehicle collisions*				
Head/neck	Yes	31	17 (55)	0.94 (0.28–3.18)
	No	16	9 (56)	

Upper limb	Yes	23	14 (61)	1.28 (0.41–4.06)
	No	24	12 (50)	

Body stem	Yes	8	4 (50)	0.77 (0.17–3.55)
	No	39	22 (56)	

Lower limb	Yes	28	18 (64)	2.48 (0.75–8.17)
	No	19	8 (42)	

Severity parameters				
Multiple injuries	Yes	32	19 (59)	1.67 (0.49–5.75)
	No	15	7 (47)	

Glasgow coma score ≤12	Yes	10	7 (70)	2.21 (0.49–9.89)
	No	37	19 (49)	

Fatal outcome	Yes	8	7 (88)	7.37 (0.83–65.66)
	No	39	19 (49)	

a Confidence interval.
